# Vertebra Plana with Paraplegia in a Middle-Aged Woman Caused by B-Cell Lymphoma: A Case Report

**DOI:** 10.1155/2012/101506

**Published:** 2012-12-23

**Authors:** Mohd. Zahid, Sohail Ahamed, Jitesh Kumar Jain, Ravish Chabra

**Affiliations:** Department of Orthopaedics, JN Medical College, Aligarh Muslim University, Aligarh, India

## Abstract

Vertebra plana is a rare presentation of spinal lymphoma. When radiological picture of a patient of paraplegia presents vertebra plana, diagnosis becomes a challenge. In a developing country like India tuberculosis should also be a consideration. Even histology sometimes fails to conclude a diagnosis. Immunohistochemistry is of immense help in clinching a diagnosis.

## 1. Introduction

Vertebra plana is a radiological diagnosis which represents flattening of a vertebral body with relatively preserved intervertebral disc space. Eosinophilic granuloma is the most common cause of vertebra plana. Other causes include metastatic disease, multiple myeloma, lymphoma, leukemia, Ewing's sarcoma, gaucher disease, Tuberculosis, and aneurysmal bone cyst.

Primary lymphoma of bone is rare. Lymphoma can occur at any age but becomes more common in sixth and seventh decades of life. Incidence of lymphoma varies greatly from region to region. For reasons that are unclear, incidence of lymphoma appears to be increasing every year [1]. In one large series primary lymphoma of bone accounted for 5 per cent of all malignant bone tumors [2]. Tumor-related spinal cord injury (SCI) represents 25% of nontraumatic SCIs and 8% of all SCI cases [3]. Most patients with spinal lymphoma have only complaint of back pain. They may also have nerve root or cord compression. In contrast to multiple myeloma which is seen in the same age group, patients with lymphoma feel otherwise healthy. Diagnosis of spinal lymphoma can be challenging. MRI findings can produce diagnostic dilemma. Even histopathological studies sometime fail to reach the correct diagnosis. Immunohistochemistry often gives clues to clinch a proper diagnosis. Understanding the tumor type for correct identification allows for treatment planning and prognosis setting. The primary treatment of lymphoma is chemotherapy. Radiation is required for local control of disease [4]. Surgical intervention is needed to relieve the symptoms of cord compression.

## 2. Case Report

The case concerns a 40-year-old woman who presented at our outpatient department with 2 years history of low back pain and lower limb weakness for 1 month. She presented to us with bladder incontinence and inability to ambulate. The bladder dysfunction had started 7 days before hospital presentation. 

On examination, she looked depressed and pale; there was no lymphadenopathy and apparent organomegaly. She had tenderness of lower thoracic spinal processes. Neurological examination of the legs showed reduced tone, and grade 1 paraplegia (MRC Scale). Knee jerks as well as ankle jerks were absent, both plantar reflexes were not elicitable, and there was sensory deficit over both lower limbs below mid-thigh level. No clinically significant past history was present. She was admitted and investigated. 

Investigations showed normal serum biochemistry apart from a mild increase of alkaline phosphatase, which was 15 KAU/L (normal 2–13). The total white cell count was within normal limit, differential count showed neutrophils 25%, lymphocytes 70%, monocytes 2%, eosinophils 3%, and basophils 0.0%, and no atypical lymphocytes were seen in blood film. The erythrocyte sedimentation rate was 24 mm/1st h.

**Figure 1:**
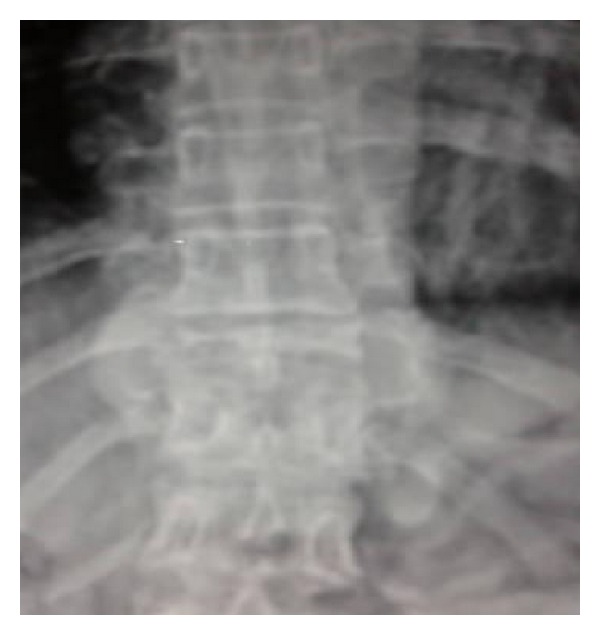
X-ray dorsolumbar spine showing vertebra plana of T10 vertebra. Disc space is well maintained.

A lumbosacral spine X-ray (Figure 1) examination showed vertebra plana of T10 vertebra with sclerosis and maintained disc space. Abdominal ultrasound was normal; no organomegaly and enlarged lymph nodes were detected. Bone marrow biopsy was done which showed normal bony trabeculae. MRI scan showed (Figure 3) vertebra plana of T10 vertebra with complete marrow replacement of vertebral body and posterior elements with associated homogenously enhanced soft tissue component in adjacent pre- and paravertebral space and in ventral and dorsal epidural spaces leading to severe cord compression and spinal canal stenosis. Although radiological picture was not in favor of tuberculosis, we started antitubercular treatment, as tuberculosis is very common in India and we have seen the cases of spinal tuberculosis with unusual presentation. However, patient did not respond to antitubercular treatment.

**Figure 2:**
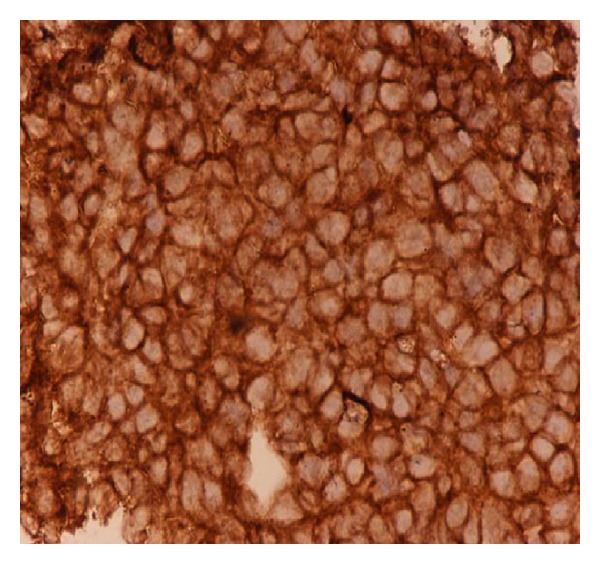
Histopathological examination of the excised tissues shows malignant small round cells with some rosette formation, suggesting a diagnosis of small round cell tumor.

**Figure 3:**
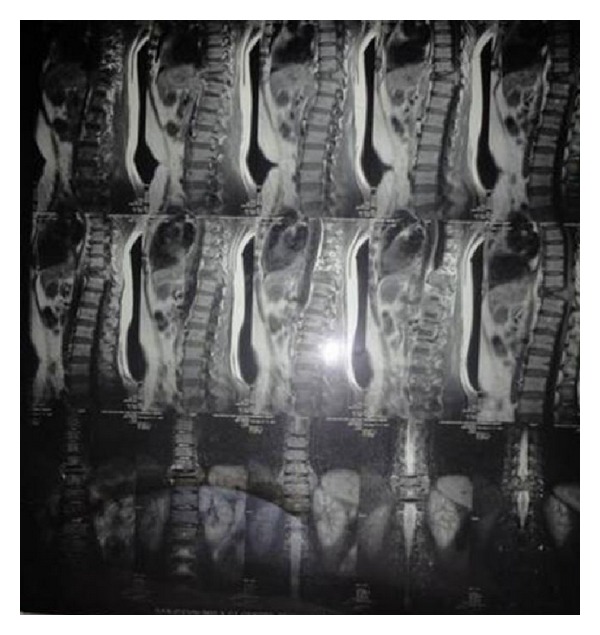
MRI of dorsolumbar spine showing complete marrow replacement of vertebral body and posterior elements with associated homogenously enhanced soft tissue component in adjacent pre- and paravertebral space and in ventral and dorsal epidural spaces leading to severe cord compression and spinal canal stenosis.

The patient was operated and anterolateral decompression of spinal cord was done to relieve symptoms of cord compression. Histopathological examination showed malignant small round cells (Figure 2) with some rosette formation. A diagnosis of small round cell tumor was suggested on basis of these findings. Immunohistochemistry examination showed positivity of tumor cells for common leukocyte antigen distinguishing it from round cell tumor. 

The patient improved remarkably after surgery. Sensory symptoms improved and motor power regained to 4/5 on hip and 5/5 on knee and ankle (MRC Scale). Bladder control could not be gained 30 days after surgery.

## 3. Discussion

 Spinal lymphomas commonly present as extradural disease, either because of an isolated deposit within the spinal canal or by the extension from an adjacent nodal mass or bone involvement. Less commonly, non-Hodgkin's lymphoma may arise in subdural space or within the spinal cord [5]. Spinal cord compression typically presents with back pain, leg numbness and tingling, radicular pain followed by extremity weakness, paresis, or paralysis. Lymphoma of spine can also be asymptomatic at presentation [6]. 

Lymphomas are a heterogeneous group of malignancies of B cells or T cells. They usually originate in the lymph nodes but may originate in any organ of the body [7]. Extranodal disease is an adverse prognostic factor, particularly the involvement of the central nervous system [6]. Between 5% and 10% of patients with nodal presentation of lymphoma may develop CNS involvement.

Histologically, lymphomas may be subdivided into non-Hodgkin lymphomas and Hodgkin lymphomas. Although secondary involvement of bones is relatively common in Hodgkin lymphoma, primary Hodgkin bone lymphoma is extremely rare. Non-Hodgkin bone lymphomas are considered primary only if a complete systemic workup reveals no evidence of extraosseous involvement. 

Histologically, the tumor consists of aggregates of malignant lymphoid cells replacing marrow spaces and osseous trabeculae. The cells contain irregular or even cleaved nuclei. The most important single procedure used to distinguish lymphoma from the other round cell tumors is the stain for leukocyte-common antigen, because lymphoid cells are the only cells that stain positively. 

Radiographically, spinal lymphoma produces a permeative or moth-eaten pattern of bone destruction or is a purely osteolytic lesion with or more commonly without a periosteal reaction. The affected vertebra can also present with an “ivory” appearance. Vertebra plana is an uncommon presentation. Because lymphoma usually does not evoke significant periosteal new bone formation, this is an important feature in differentiating it from Ewing's sarcoma.

The intermediate or high histological type of non-Hodgkin's lymphoma and the presence of an underlying immune deficiency are the most significant risk factors for secondary CNS involvement [8]. CNS presentations may include spinal cord compression, leptomeningeal spread, or intracerebral mass lesions. In addition, other mechanisms of neuropathy should be considered, such as the effects of chemotherapy. Spinal cord compression is a rare presentation of non-Hodgkin's lymphoma.
